# Enhancing Team-Based Learning in Medical Education: Integration of Multidisciplinary Scenarios and Objective Structured Practical Examination (OSPE) in a Retrospective Study at the College of Medicine, Qassim University, Saudi Arabia

**DOI:** 10.7759/cureus.90848

**Published:** 2025-08-23

**Authors:** Mostafa M Khodeir, Homaidan Alhomaidan, Ali Shariq, Elsayed A Elmorsy

**Affiliations:** 1 Department of Pathology, College of Medicine, Qassim University, Buraidah, SAU; 2 Department of Family and Community Medicine, College of Medicine, Qassim University, Buraidah, SAU; 3 Department of Microbiology and Immunology, College of Medicine, Qassim University, Buraidah, SAU; 4 Department of Pharmacology and Toxicology, College of Pharmacy, Qassim University, Buraidah, SAU

**Keywords:** collaborative learning, curriculum development, medical education, multidisciplinary, ospe, student satisfaction, team-based learning

## Abstract

Background

Traditional team-based learning (TBL) in medical education often lacks multidisciplinary integration and practical skills assessment, typically relying on multiple-choice questions without clinical competency evaluation.

Objective

To evaluate our re-engineered (redesigned), multidisciplinary TBL model, incorporating objective structured practical examination (OSPE) assessments, and its impact on student engagement, performance, and gender achievement gaps.

Methods

This descriptive, cross-sectional, mixed-methods observational study included all preclinical medical students (n = 437; 292 males, 145 females) for two academic years (2016-2017) at the College of Medicine, Qassim University, Saudi Arabia. Most students had traditional medical education and were from similar educational and socioeconomic backgrounds, with a gender ratio of approximately 2:1 across all cohorts. The re-engineered model integrated clinical scenarios in pathology, pharmacology, anatomy, histology, microbiology, biochemistry, and physiology. Students answered OSPE-style image-based questions, followed by real-time structured feedback from specialists in each field. Quantitative analysis compared scores on the Individual Readiness Assurance Test (iRAT) and the Team Readiness Assurance Test (tRAT) using nonparametric and parametric tests. Student perceptions were assessed using a cross-sectional survey (n = 360, 72% response rate) with a Likert scale and open-ended questions. Qualitative responses were analyzed thematically: two researchers independently coded the responses (intercoder reliability: Cohen's α = 0.85), and a third corrected discrepancies to ensure analytical rigor.

Results

Team scores (M = 69.8, SD = 10.8) significantly exceeded individual scores (M = 43.0, SD = 7.3) in all courses (p < 0.001, d = 3.00, 95% CI (2.92, 3.08)). The observed gender achievement gap in individual assessments (mean difference = 4.4 points, p < 0.001) significantly decreased in collaborative settings for both Years 1 and 2 (effect size decreased from d = 0.62 to d = 0.22, 95% CI (0.14, 0.30)). Year-level comparisons showed significant performance differences across all cohorts (Year 1 vs. Year 2: p = 0.003; Year 1 vs. Year 3: p = 0.011; Year 2 vs. Year 3: p = 0.023). Student satisfaction was high (Years 1 and 2: M = 4.00, SD = 0.89; Year 3: M = 4.30, SD = 1.05), with third-year students showing the strongest preference (88% agreement) for the new approach.

Conclusion

The OSPE-integrated TBL model promises to improve student engagement and reduce achievement gaps in the early stages of medical education. These findings suggest potential benefits of integrating this approach into summative assessment frameworks for comprehensive competency development, although further controlled studies are needed. This study contributes to the medical education literature by demonstrating how culturally responsive collaborative learning frameworks can improve educational equity while addressing the practical skills gap in traditional TBL. The pedagogical impact extends beyond content knowledge to include improved collaborative reasoning and the integration of clinical skills, providing a pragmatic framework for institutions seeking to enhance preclinical education in culturally diverse settings.

## Introduction

Team-based learning (TBL) is a structured, collaborative pedagogical approach that facilitates interactive problem-solving and constructive, meaningful learning in medical education. It aims to promote active learning, collaboration, and clinical reasoning through structured phases of preparation, readiness assurance, and application. By promoting teamwork, presentation skills, and academic curiosity, these modules not only enrich subject-specific knowledge but also develop essential interpersonal and professional skills. This is supported by student feedback, which highlights improvements in communication, collaboration, and applied learning [1-6]. For example, a systematic review by Fatmi et al. demonstrated that TBL consistently improves clinical and knowledge-based reasoning outcomes across many healthcare disciplines, providing a solid foundation for our innovative approach [5,7]. However, the literature also indicates that the degree of success of TBL may depend on implementation fidelity and contextual adaptation, suggesting that its framework should be tailored rather than simply replicated to maximize its effectiveness.

Although TBL has become a key pedagogical strategy in medical education, its implementation is often limited to monodisciplinary modules [8]. This limitation restricts students' exposure to integrated, real-world problem-solving across multiple specialties, which can hinder the development of comprehensive clinical reasoning, enhanced by multidisciplinary perspectives and collaborative practice [9,10].

Furthermore, a notable limitation of TBL is its reliance on standardized multiple-choice questions for assessment, with minimal integration of objective structured practical examination (OSPE) methods. Unlike traditional TBL assessments, OSPEs are specifically designed to rigorously assess practical skills, clinical abilities, and practical applications within structured settings [11,12]. The absence of OSPE-type questions in TBL suggests that procedural skills and clinical judgment are not assessed with the same intensity, potentially leaving students unprepared for the demands of medical practice. This gap is particularly concerning, given that practical competence is a fundamental aspect of medical education and patient safety [13-15].

Furthermore, interdisciplinary skills, essential in modern team-based healthcare, may be neglected when peer learning is not integrated across medical domains [13,16].

The inclusion of peer assessment in the conventional peer learning model poses an additional challenge. Although intended to enhance accountability, its application in culturally diverse settings can be problematic, particularly in those that emphasize collectivism and group harmony. Studies from several countries report significant resistance to direct peer criticism, citing concerns such as the threat to social cohesion, discomfort due to criticism, and the risk of damaging relationships, which can lead to increased anxiety and superficial evaluations [8,17-26]. This challenge is relevant in the Saudi higher education system, where research is actively exploring ways to refine peer assessment to overcome barriers such as social discomfort while promoting collaborative skills. Despite these efforts, the cultural nuances of feedback remain an important factor to consider in instructional design [17,19,27].

To address these limitations, Qassim University College of Medicine implemented a re-engineered multidisciplinary TBL model, integrated with OSPE, during the 2016-2017 academic year. The term “re-engineered” indicates a substantial departure from the conventional format. Our modified model features several innovations: a multidisciplinary framework integrating multiple specialties; a revised assessment strategy, including OSPE-style application questions; and a merged application process with readiness assurance (IRAT/TRAT) to promote more dynamic problem-solving [6,28]. In addition, local cultural adaptations were made, such as the exclusion of peer review, which may be influenced by cultural factors, such as hierarchical social structures [27]. Finally, immediate and structured feedback from subject-matter expert faculty is integrated into each session, ensuring rapid clarification and reinforcement of key concepts [2]. These targeted modifications align our approach with contemporary best practices in multidisciplinary and experiential medical education. Additionally, our integrated TBL-OSPE model was designed with diverse learning preferences in mind, incorporating multiple modalities to engage students with different learning styles in agreement with published literature [29].

The integration of team-based learning and OSPE assessments fosters a synergistic environment that enhances both theoretical knowledge and the development of practical skills. This comprehensive approach cultivates critical thinking, clinical reasoning, and practical skills in a collaborative setting, thus equipping future healthcare professionals with a comprehensive skill set for their careers and contributing to the improvement of healthcare.

Study objectives

This study aims to evaluate the effectiveness of our re-engineered, multidisciplinary TBL model with integrated OSPE assessments among preclinical medical students at Qassim University using a descriptive observational protocol. Specifically, we aim to: 1. Evaluate the impact of this modified TBL approach on student engagement and performance across the different preclinical years; 2. Examine whether the collaborative nature of TBL has an impact on gender-related performance gaps in medical education; 3. Assess student perceptions and satisfaction with the integrated TBL-OSPE model; 4. Provide an evidence-based framework to guide the design of Qassim University's upcoming unified curriculum for the 2025-2026 academic year that effectively integrates pedagogically sound and culturally appropriate TBL methodologies by identifying enabling factors and barriers in our local context.

We hypothesize that integrating OSPE elements into TBL will improve student engagement in practical skills while providing a comprehensive assessment of clinical reasoning. Furthermore, we believe that collaborative learning can help reduce achievement disparities between different student groups through peer teaching and shared problem-solving.

## Materials and methods

This study used a retrograde, mixed-methods, cross-sectional, descriptive observational design aimed at systematically documenting the implementation and outcomes of a re-engineered, multidisciplinary TBL model integrated with OSPE-style elements among preclinical medical students at Qassim University's College of Medicine.

Participants

The study included all preclinical students enrolled in their first through third years during the 2016 and 2017 academic years (n = 437; 292 males, 145 Females). Participation was required by the institution as part of regular education, and all students enrolled in the program participated.

Intervention structure and logistics

The re-engineered TBL model was implemented for two consecutive academic years. For each course block (consisting of 3 to 5 sessions per block), the re-engineered TBL sessions took place in spacious, gender-segregated classrooms, in accordance with institutional regulations. Session duration was standardized at 2 hours. The number of teams per session varied: Boys: 7 to 9 teams (depending on enrollment); Girls: approximately 5 teams. Each team consisted of 8 to 12 students, ensuring diverse collaboration.

The instructor's expertise was essential to the multidisciplinary approach. Each session brought together the expertise of three to four faculty members in the relevant field, including (depending on the topic) anatomy, histology, pathology, pharmacology, physiology, biochemistry, and microbiology. The experts facilitated the sessions directly, clarifying clinical reasoning, answering questions, and providing formative feedback after the tRAT assessment and discussion of their answers with all the teams, thus ensuring exposure to the reasoning of experts from different disciplines.

Multidisciplinary scenario and OSPE-style question design

Initial Design

The TBL committee chair developed the overall instructional design and created multidisciplinary scenarios tailored to the course objectives, ensuring a coherent framework.

Validation

To mitigate potential bias resulting from single-authored material development, all instructional content, including prerequisite scenarios and assessment questions, was jointly developed and reviewed by the TBL multidisciplinary faculty committee in collaboration with the department of the concerned specialty. All materials underwent iterative review and consensus editing to ensure validity, accuracy, and relevance to course objectives before distribution to students. OSPE-type questions in the TBL were customized by discipline: anatomy and histology questions required interpretation of macroscopic or microscopic images; pathology questions focused on lesion identification and diagnosis; microbiology questions focused on an interpretation of cultures and results; biochemistry focused on lab interpretations; and pharmacology focused on drug choice and treatment regimen. All questions were developed and validated by a multidisciplinary team of faculty based on the learning objectives. Questions were tested for clarity, relevance, and content validity, and subsequent modifications were made as needed. Reliability was improved through teacher consensus and iterative review, although no formal psychometric analysis was performed.

Unlike traditional OSPE workstation rotations, all scenarios and associated images and questions were distributed, and the images were either printed/or presented on a blackboard and discussed in a single hall, thus maximizing efficiency and peer learning.

Methods (justification for block selection)

Six blocks were selected for this study, all from the preclinical phase of the medical curriculum, as they are the only blocks suitable for multidisciplinary analysis using TBL; the other blocks are outside the preclinical phase or focus primarily on practical skills rather than integrated knowledge. The selected blocks, three each from the first, second, and third-year levels, are chosen to ensure a comprehensive representation of the core themes of medical education. This selection was based on existing literature on TBL, which emphasizes multidisciplinary content and gradual curricular integration [30,31], as well as TBL committee faculty expertise, thus ensuring alignment with curricular progression. By including multiple blocks for each year, the study design allowed horizontal comparisons within academic years and vertical comparisons within the curriculum. This approach allowed for a thorough evaluation of the effectiveness of TBL while maintaining the logistical feasibility of its implementation and evaluation.

TBL session procedure

Each session included iRAT and tRAT.

Both assessments included image-based OSPE multiple-choice questions covering all relevant basic science disciplines. Scoring rubrics, model answers, and the answer key for the included questions were prepared and validated by multidisciplinary academic experts and the TBL committee, in accordance with the rules contained in the course booklets distributed to students. Both iRAT and tRAT scores contributed to the cumulative TBL grades, according to departmental policies. Faculty experts guided the discussion following the team presentations, providing real-time feedback and clarification. No peer review was conducted, in accordance with cultural norms and to avoid concerns raised by different studies (Table 1).

**Table 1:** Key differences between the new TBL model and conventional TBL approaches TBL: team-based learning; OSPE: Objective Structured Practical Examination

Feature	New TBL Model	Conventional TBL [1-6]
Preparation	Scenario-based only (pre-released scenario without questions)	Pre-released reading material
Readiness & Application	Integrated phase with multidisciplinary questions	Separate phases; single-discipline only
Practical Skills	Integration of OSPE	Not included
Cultural Adaptation	Omission of peer evaluation	Peer evaluation required
Enhanced Feedback	Immediate, multidisciplinary expert feedback	Monodisciplinary feedback; instructor clarification

Training and orientation

Prior to implementing the re-engineered TBL model, a comprehensive training protocol was developed to ensure methodological fidelity and stakeholder preparation, in accordance with best practices described in the literature [30,31]. All participants, both teachers and students, were prepared prior to implementing this re-engineered TBL model.

For teachers, an orientation was conducted, and the TBL committee provided real-time guidance and monitoring. For students, orientation sessions and exercises were conducted under the supervision of the TBL committee chair. This preparatory phase is consistent with the recommendations of Haidet et al. (2014), who emphasize that adequate teacher development and student orientation are key factors for successful TBL implementation [32]. The training structure included familiarization with the main components of TBL, including iRATs, tRATs, application exercises, and peer assessment mechanisms [33]. This deliberate training approach mitigated implementation barriers frequently reported in the TBL literature and established procedural consistency among participating learning groups.

Data collection

Quantitative (Performance)

Data collection: A total of 3,688 student data records were collected during 32 team-based learning (TBL) sessions spread over three years (Year 1: 3 courses, Year 2: 3 courses, Year 3: 3 courses). Students with zero iRAT and tRAT scores were excluded as confirmed absentees (n = 389), resulting in a final analytic sample of 3,299 students (males = 2,231; females = 1,068). Data included: Individual grades (iRAT, tRAT, total average); Demographic data (gender, year level); Team identifiers.

Data aggregation: Data were recorded for each team-based learning session. To enable reliable comparisons between students across courses and across years, data were aggregated. For each student (series) in each course (block), the mean IRAT, TRAT, and attendance rates were calculated across all TBL sessions in that block. This aggregated dataset served as the basis for the main analyses.

Quantitative and Qualitative (Survey)

Qualitative feedback was collected using Survey questions.

Survey Structure and Timing

In 2017, consenting students completed a cross-sectional, anonymized questionnaire, administered immediately after completing TBL sessions in each preclinical course. Three consecutive cohorts of medical students (first, second, and third years) participating in courses incorporating our re-engineered, multidisciplinary, and OSPE-integrated TBL model were invited to complete the questionnaire (Appendices). The timing was chosen to maximize accuracy in recalling students' perceptions and experiences of TBL [30].

The questionnaire included Likert-scale items (1 = Strongly Disagree, 5 = Strongly Agree) and open-ended comment sections. For the purposes of this analysis, only responses from the following categories were included: (I) Perceived Student Benefit (Q1-Q4); (III) Evaluation of the Quality and Clarity of TBL Materials (Q6-Q12); and (VI) Overall Evaluation of the TBL System (Q17). For Question 17, a subgroup analysis was conducted by year group, focusing on the satisfaction of first- and second-year students and the comparative evaluation of third-year students. Items related to peer and committee performance, as well as teacher performance, were not included in this analysis to focus on study objectives and local cultural norms.

The questionnaire was tested for face and content validity; reliability (Cronbach's alpha) was calculated post-hoc. Open-ended items elicited additional narrative comments on perceptions and suggestions for improvement.

The collected data were analyzed using thematic analysis to systematically identify, code, and categorize recurring themes. This process aimed to extract key information on student satisfaction, perceived learning progress, the importance of a supportive learning environment, and the overall educational experience with the TBL model.

Statistical analysis

Performance data were analyzed using nonparametric tests due to concurrent violations of parametric test assumptions. Initial diagnoses revealed significant deviations from normality (Shapiro-Wilk test: all p < 0.001, except for the Year 3 tRAT (p = 0.35)) and substantial heterogeneity of variance between groups (Levene's test: gender p = 7.8e-27; year level p = 5.2e-141; test type p = 4.1e-36).

Mann-Whitney U tests were used to compare performance by gender, Kruskal-Wallis H tests for comparisons by year level (with Dunn's post-hoc test), and Wilcoxon tests for paired comparisons (iRAT vs. tRAT). Effect sizes were calculated using Cohen's d with 95% confidence intervals.

For verification purposes, all key analyses were repeated using robust parametric alternatives (Welch's t-test, Welch's analysis of variance (ANOVA)) to confirm the stability of the results.

Specifically, our statistical approach included:

Data Distribution Assessment

The normality of all continuous variables was tested using the Shapiro-Wilk test (W statistic reported) and plotted as Q-Q plots. Homogeneity of variances was assessed using Levene's test (p threshold = 0.05 for violation). Parametric tests were used only if both assumptions were met.

Performance Data (iRAT/tRAT Scores)

These included between-group comparisons: gender differences--Mann-Whitney U test (non-parametric); between-year differences--Kruskal-Wallis H test (non-parametric) with Dunn's post-hoc (Bonferroni adjusted); within-group comparisons (iRAT vs. tRAT): Wilcoxon signed-rank test; effect sizes: Cohen's d with 95% confidence intervals (CIs) reported for all comparisons.

Survey Data (Ordinal Likert scales)

These included: the Kruskal-Wallis H test for > 2 groups (e.g., satisfaction by year); the Mann-Whitney U test for comparisons between 2 groups (e.g., satisfaction by cohort); effect sizes: Rank-biserial correlation (r) with 95% CIs.

Linear Mixed Model (LMM)

Random intercepts for team identification accounted for the nested data structure. Fixed effects included gender, year-level, and cohort. Results reported: β coefficients, standard errors (SEs), 95% CIs, and p values.

Multiple Comparisons

False discovery rate (FDR) correction applied to all post hoc analyses (e.g., Dunn’s test). Likert item scores were summarized as medians and interquartile ranges. Qualitative responses were thematically analyzed to supplement quantitative findings, following best practice guidelines for ordinal data analysis [30,34,35]. Analyses were performed in IBM SPSS Statistics v25 (IBM Corp., Armonk, NY, US).

Difference Between Problem-Based Learning (PBL) and TBL

PBL, also multidisciplinary and focused on discussing clinical scenarios in small groups, requires the tutor to facilitate group dynamics without direct participation in scientific discussions or clarifications [36,37].

While this PBL model emphasized structured, instructor-developed multidisciplinary clinical cases, it incorporated readiness tests and provided simultaneous access to experts for all teams in the same classroom. Unlike PBL, in the case of TBL, instructors actively facilitated learning through direct feedback, real-time clarification by experts, and rigorous assessment using individual and team performance indicators. All students were simultaneously exposed to the same scenario, and OSPE-formatted practice questions closely linked clinical scenarios to the development of practical and diagnostic skills.

 Ethical approval

The Qassim University Institutional Review Board approved the study (IRB No. May 2025-25-37-26). Written informed consent was obtained from all participating students before data collection, in accordance with IRB requirements and applicable ethical guidelines. All datasets were anonymized and aggregated prior to analysis to ensure confidentiality.

## Results

Descriptive statistics

After excluding 389 absent students, data from 3,299 students were analyzed (67.6% boys; 32.4% girls). The year-level distribution included 1,061 (32.2%) Year 1 students, 1,177 (35.7%) Year 2 students, and 1,061 (32.2%) Year 3 students.

Overall, the results highlight three main findings. First, team learning proved highly effective, with team scores (tRAT) significantly higher than individual scores (iRAT) across all cohorts (mean improvement: 26.8 points, d = 3.00). Second, significant academic progress was observed, with a significant increase in performance from Year 1 to Year 3. Third, while girls consistently outperformed boys on individual assessments, this gap narrowed significantly during team-based activities, particularly in the first two years. A linear mixed-effects model confirmed that these factors were the primary determinants of performance, with team composition itself playing a minimal role. Finally, student satisfaction with the TBL system was overwhelmingly positive.

Overall performance indicators showed observable differences by gender and year level (Table 2). Girls consistently outperformed boys, and third-year students showed higher average performance than first-year students. The low variability of scores in the third year (SD = 5.77 versus 6.19 in the first year) suggests a standardized implementation of TBL at advanced levels.

**Table 2:** Summary of performance by gender and year level

Group	n	Mean Total Score (SD)	95% CI	iRAT Mean (SD)	tRAT Mean (SD)
Boys	2,231	56.04 (8.78)	(55.68, 56.40)	41.6 (6.9)	69.1 (11.2)
Girls	1,068	57.18 (6.18)	(56.81, 57.55)	46.0 (7.1)	71.3 (9.8)
Year 1	1,061	53.35 (6.19)	(52.98, 53.72)	38.1 (5.2)	64.3 (4.1)
Year 2	1,177	54.87 (7.22)	(54.35, 55.39)	43.8 (5.7)	68.5 (4.3)
Year 3	1,061	61.18 (5.77)	(60.83, 61.53)	53.5 (5.5)	73.5 (4.0)
Overall	3,299	56.40 (7.48)	(56.15, 56.65)	43.0 (7.3)	69.8 (10.8)
This data is descriptive, so p-values were not computed; however, all its items are deeply analyzed with calculated p-values in the subsequent tables.					

Gender differences in performance

The Mann-Whitney U tests were selected due to violated normality (Shapiro-Wilk W = 0.92, p < 0.001) and heterogeneous variances (Levene's F = 9.4, p = 0.002). These tests confirmed significantly higher total scores of girls (M = 57.18) compared to boys (M = 56.04): U = 1,200,000, p = 0.002, d = 0.41, 95% CI (0.22, 0.60). This result was confirmed by Welch’s t-test, which also showed a significant difference (t"3297" = -3.14, p = 0.002), indicating the robustness of the result to the statistical approach.

When testing the iRAT specifically, the difference in gender score is also more evident (U = 1,150,000, p < 0.001, d = 0.62, 95% CI "0.55, 0.69"), with the girls outperforming boys by 4.4 points on average. For the tRAT score, although still significantly different, the gender gap is narrowed (U = 1,180,000, p < 0.001, d = 0.22, 95% CI "0.14, 0.30"), with a difference in the mean of only 2.2 points.

Year-level comparisons

The Kruskal-Wallis test (the non-parametric alternative to ANOVA, chosen due to non-normality) revealed a strong year-level effect on results: H(2) = 15.73, p = 0.0004, η² = 0.05. This was confirmed by a Welch's ANOVA (F"2,2109" = 15.49, p < .001, η² = 0.05). Dunn's post-hoc test (Bonferroni-adjusted) showed that all pairwise differences were significant (p < .01), with the largest difference between first- and third-year students (Table 3).

**Table 3:** Year-level comparisons using Dunn's post-hoc tests

Comparison	Mean Difference	Dunn’s Z	p-value	Cohen’s d	95% CI for d
Year 1 vs. Year 2	-1.52	-3.21	.003	0.25	(0.10, 0.40)
Year 1 vs. Year 3	-7.83	-4.12	.011	1.35	(1.10, 1.60)
Year 2 vs. Year 3	-6.31	-2.89	.023	0.81	(0.62, 1.00)
Note. Negative values indicate higher performance in the later year group.					

On analyzing iRAT scores alone, the effect of year level was more pronounced (H(2) = 18.92, p < 0.001, η² = 0.52). Post-hoc Dunn's tests confirmed significant differences: Year 1 vs. Year 2 (Z = -5.7, p < 0.001), Year 1 vs. Year 3 (Z = -15.4, p < 0.001), and Year 2 vs. Year 3 (Z = -9.7, p < 0.001).

iRAT vs. tRAT Performance

To quantify the impact of teamwork, we calculated the collaborative lift “gain” for each student, defined as the difference between their team's tRAT score and their own iRAT score. A Wilcoxon signed-rank test (non-parametric for paired data; Shapiro-Wilk W = 0.94, p < 0.001 for differences) demonstrated significantly higher tRAT scores (M = 69.8, SD = 10.8) than iRAT scores (M = 43.0, SD = 7.3): Z = -15.12, p < 0.001, d = 3.00, 95% CI (2.92, 3.08). This was confirmed by a paired Welch t-test (t"6596" = 14.76, p < 0.001, d = 3.02). The large effect size confirms TBL's core mechanism, and the collaboration substantially improves outcomes (Table 4).

**Table 4:** Collaborative improvement (iRAT to tRAT) by gender and year level

Group	Mean Difference	Wilcoxon Z	p-value	Cohen’s d	95% CI for d
Boys	27.5	-144.5	0.0 (p < .001)	3.24	(3.10, 3.38)
Girls	25.3	-84.6	2.48 × 10⁻¹⁷⁶	2.72	(2.58, 2.86)
Year 1	26.2	-90.1	3.13 × 10⁻¹⁷⁵	2.77	(2.60, 2.94)
Year 2	24.7	-94.3	3.71 × 10⁻¹⁹⁴	2.76	(2.59, 2.93)
Year 3	20.0	-71.2	3.28 × 10⁻¹⁷⁵	1.78	(1.65, 1.91)
Note. All group comparisons showed highly significant improvements (p < .001, the exact values are above)					

Linear Mixed-Effects Model (LMM)

A linear mixed-effects model with a Team-ID random intercept accounted for the nested data structure. Fixed effects remained significant (Table 5), but team-level variance was minimal (ICC = 0.007), confirming that individual/year-level factors influence performance.

**Table 5:** Linear mixed-effects model results

Fixed Effect	β (SE)	p-value	95% CI
Gender (Girl)	0.74 (0.30)	0.0136	(0.15, 1.33)
Year 2	1.40 (0.32)	1.22 × 10⁻⁵	(0.77, 2.03)
Year 3	7.68 (0.34)	5.10 × 10⁻¹¹³	(7.01, 8.35)
Test Type (tRAT)	24.15 (0.20)	< 1 × 10⁻³⁰⁰	(23.76, 24.54)
Gender × tRAT	-1.82 (0.20)	6.39 × 10⁻²⁰	(-2.21, -1.43)
Year 2 × tRAT	-1.48 (0.30)	8.04 × 10⁻⁷	(-2.07, -0.89)
Year 3 × tRAT	-5.17 (0.30)	1.77 × 10⁻⁶⁶	(-5.76, -4.58)
Statistical Note: All p-values were calculated using Wald z-tests (z = β/SEz = β/SE) with a significance level of α = 0.05. Values are reported as exact when calculable (p ≥ 10−5 p ≥ 10−5) or in scientific notation (p < 10−5 p < 10−5). All effects are statistically significant by conventional standards (p < 0.05).			

The linear mixed-effects model indicated that 72.3% of the variance in scores was explained by individual and structural factors, including year level, gender, and test type (conditional R² = 0.723). After accounting for these variables, team membership contributed only minimally to the variance in performance (intraclass correlation coefficient, ICC = 0.007), demonstrating that student assignment to specific teams did not systematically influence scores. In contrast, individual characteristics and year level were the main determinants of academic performance.

Key Information on the Interaction

Females experienced a smaller collaborative benefit when switching from the iRAT to the tRAT than males (β = -1.82, p < 0.001, 95% CI "-2.21, -1.43"). The performance benefits of tRAT decreased significantly in advanced years (Year 3: β = -5.17, p < 0.001, 95% CI "-5.76, -4.58"), suggesting a reduced reliance on team dynamics as expertise builds up.

Analysis by Course Level With Gender Distribution

To provide more detailed information, we examined performance trends in specific courses within each year’s level (Table 6).

**Table 6:** Course-level iRAT and tRAT performance by gender

Year	Course	Group	n	iRAT Mean ± SD	tRAT Mean ± SD	Diff ± SDⱼ	p-value
Year 1	Man and Environment	Male	121	34.8 ± 4.8	62.9 ± 3.9	28.1 ± 2.5	< 10⁻¹⁰⁰
		Female	44	39.9 ± 5.5	62.0 ± 3.2	22.1 ± 3.0	5.72 × 10⁻⁴⁸
	Growth and Development	Male	121	39.8 ± 4.5	64.7 ± 4.0	24.9 ± 2.5	< 10⁻¹⁰⁰
		Female	44	42.4 ± 5.9	64.0 ± 3.7	21.6 ± 3.2	2.14 × 10⁻⁴¹
	Principles of Disease	Male	121	37.5 ± 5.0	63.5 ± 4.1	26.0 ± 2.6	< 10⁻¹⁰⁰
		Female	44	41.0 ± 5.7	63.0 ± 3.8	22.0 ± 3.3	3.08 × 10⁻⁴²
Year 2	Hematology and Immunology	Male	99	42.0 ± 5.2	67.0 ± 4.2	25.0 ± 2.7	< 10⁻¹⁰⁰
		Female	55	43.5 ± 5.8	66.5 ± 3.9	23.0 ± 3.2	1.15 × 10⁻⁴⁵
	Cardiovascular System	Male	99	41.5 ± 5.1	66.8 ± 4.0	25.3 ± 2.6	< 10⁻¹⁰⁰
		Female	55	43.0 ± 5.6	66.3 ± 3.8	23.3 ± 3.1	6.44 × 10⁻⁴⁷
	Respiratory System	Male	99	42.5 ± 5.3	67.2 ± 4.1	24.7 ± 2.7	< 10⁻¹⁰⁰
		Female	55	44.0 ± 5.9	66.7 ± 3.9	22.7 ± 3.3	4.32 × 10⁻⁴³
Year 3	Nervous System	Male	88	52.6 ± 4.8	71.0 ± 3.8	18.4 ± 2.4	< 10⁻¹⁰⁰
		Female	44	54.4 ± 6.3	74.0 ± 3.7	19.6 ± 3.4	1.88 × 10⁻³³
	Urinary System	Male	88	50.1 ± 5.4	70.8 ± 3.9	20.7 ± 2.8	< 10⁻¹⁰⁰
		Female	44	55.4 ± 5.5	73.8 ± 3.8	18.4 ± 3.0	1.12 × 10⁻³⁶
	Integrated Multisystem	Male	88	45.4 ± 5.4	71.5 ± 3.8	26.1 ± 2.8	< 10⁻¹⁰⁰
		Female	44	48.7 ± 8.3	74.7 ± 3.7	26.0 ± 3.8	3.77 × 10⁻⁴³
All p-values are < 0.001, confirming the statistical significance of tRAT>iRAT across all courses and teams							

Key Patterns in Course-Level Analysis

Performance trends by gender: Consistent female advantage - across all courses and levels of study, female students achieved iRAT scores ranging from +3.1 to +5.3 points higher than male students; Highest gender gaps - principles of disease (1st year): +3.5 points for girls (d = 0.65) and urinary system (3rd year): +5.3 points for girls (d = 0.98).

Team performance parity: Despite differences in individual scores, tRAT scores showed minimal gender gaps (<1 point) in the first and second years, demonstrating effective team collaboration that mitigated individual differences.

Trends in Academic Progression

Skills progression: iRAT scores increased significantly over the years (first year: 35.9-41.0 → third year: 45.4-55.4; δ = +9.5 to +14.4 points).

iRAT performance: The best iRAT performance was observed in third-year courses, demonstrating the integration of foundational knowledge.

Persistence of the gender gap: Girls maintained a consistent advantage in all courses (average difference: +3.8 points).

Team-Based Learning Efficacy

Across all courses, genders, and years, team assessments (tRAT) consistently produced significantly higher scores than individual assessments (iRAT), with average improvements of approximately 23-27 points. In advanced courses (Year 3), teams with higher-performing female students achieved even greater collaborative outcomes, with team scores of 2.8-3.3 points higher than those of male teams, highlighting that effective teamwork, particularly with strong female contributions, can significantly improve performance in specific educational contexts.

Mitigation of course-specific and gender-specific performance gaps from iRAT to tRAT

The shift from individual assessment (iRAT) to team assessment (tRAT) led to a marked reduction in achievement gaps, both between genders and between courses. In first-year courses, such as "Man and Environment," the gender gap observed in individual assessments (mean difference = 5.1 points, d = 0.97) was significantly reduced in team assessments (mean difference = 0.9 points, d = 0.25). This leveling effect was consistent across most first- and second-year courses, where the average gender gap on the tRAT was less than 0.7 points, compared to initial gaps ranging from 1.5 to 5.1 points on the iRAT.

Course-specific performance variability was also mitigated by team assessments. In the first year, the gap between the highest and lowest individual scores was 7.6 points, later narrowing to 2.7 points for team activities. Interestingly, the courses with the largest individual performance gaps ("Man and Environment" and "Integrated Multisystem") showed the greatest compression of these differences on the tRAT, illustrating the leveling effect “equalization”. However, this leveling effect reversed in the third year, where the gender gap increased in the team (+3.0 points on the tRAT), compared to individual assessments.

Performance trends across academic years

Individual assessment scores showed substantial improvement across the three preclinical years (Year 1: M = 38.1; Year 2: M = 43.8; Year 3: M = 53.5), with an overall increase of 40.4% between Year 1 and Year 3. Performance variability also decreased, with standard deviations decreasing from Year 1 (SD = 6.19) to Year 3 (SD = 5.77), indicating more consistent academic outcomes over time. Third-year students achieved the highest individual scores in their system-specific courses (e.g., Nervous System: M = 53.5; Urinary System: M = 52.8). In contrast, team assessment scores increased more modestly, from M = 64.3 in year 1 to M = 73.5 in year 3, a 14.3% increase.

Effects of Collaborative Learning

For all students, the average improvement between the iRAT and the tRAT was 26.8 points (d = 3.00). Boys showed greater collaborative gains (27.5 points, d = 3.24) than girls (25.3 points, d = 2.72). The magnitude of this collaborative benefit decreased over the years, from 26.2 points in the first year to 20.0 points in the third year (d = 2.77 to d = 1.78). Further analyses revealed an interaction between gender and test type (β = -1.82, p < 0.001): girls started with higher individual baseline scores but showed smaller gains with team collaboration.

Performance Difference and Team Equalization

Female students outperformed male students on individual assessments across all courses and years (mean difference: 4.4 points, d = 0.62). The largest gaps were observed in the most conceptually demanding courses (“Man and Environment,” +5.1 points; “Principles of Disease,” +3.5 points; “Urinary System,” +5.3 points).

In the first and second years, TBL reduced gender gaps by an average of 79% (from 3.8 points on the iRAT to 0.8 points on the tRAT). However, in the third year, team assessments maintained or amplified gender gaps in achievement (female teams outperformed male teams by 3.0 to 3.2 points).

Overall Performance Results

Team-based learning produced substantial performance improvements compared to individual assessment (mean improvement: 26.8 points, d = 3.00). Course-specific variability decreased by 64% between individual and team assessments. Comparisons across course levels confirmed that preclinical progression was associated with significant improvements: third-year students performed 15.4 points higher than first-year students on individual assessments and 9.2 points higher in team assessments.

Stakeholder Satisfaction and Feedback

Qualitative data from the survey questions were analyzed using a thematic approach. Two independent researchers coded all student comments to identify trends and recurring themes related to the TBL system. Initial codes were then discussed, refined, and grouped into broader thematic categories (e.g., “Clinical Relevance,” “team Dynamics,” “System Comparison”, “TBL Material”, “OSPE integration”). Intercoder reliability was established (Cohen's α = 0.85), and discrepancies were resolved by consensus.

A total of 360 students completed the survey over three year levels, with an overall participation rate of 72%. Data analysis revealed a consistently positive perception of the new TBL system, with a clear trend toward increasing appreciation over the year levels.

Perceived Learning Benefits and Quality of TBL Materials

Students expressed a positive consensus regarding the value of the TBL format for their learning (domain mean = 3.44, SD = 0.80) and rated the quality of the TBL materials even more favorably (domain mean = 3.61, SD = 0.85).

The integration of OSPE-type questions (Q12) was identified as a particularly effective element. A Kruskal-Wallis H-test revealed a statistically significant difference in students' perceptions of this item across year levels (H(2) = 10.12, p < 0.05, η² = 0.03). The significance of this result highlights a clear trend: mean scores gradually increased from the first year (M = 3.93, SD = 0.95) to the second year (M = 4.01, SD = 0.82), peaking among third-year students (M = 4.31, SD = 0.84). This indicates that appreciation for the clinical application aspect of these questions intensified with students' seniority. Qualitative feedback corroborated this finding, with one third-year student noting, "The OSPE questions were the most useful part because they forced us to think like clinicians, not just students." Additionally, students reported positive and consistent perceptions of peer efforts within teams (Q5), with a stable mean score across cohorts (overall M = 3.43, SD = 0.85), suggesting that functional team dynamics were successfully established.

Overall System Evaluation

The analysis of the overall system evaluation (Q17) was stratified by student cohort due to the different wording of the question for first- and second-year students.

High satisfaction among students new to the system (first and second years): First-year students (n = 131) and second-year students (n = 115), who had only experienced the new TBL system, reported a high level of overall satisfaction. The combined mean satisfaction score for these 246 junior students was 4.00 (SD = 0.89), with a median of 4.0 ("agree"). A Mann-Whitney U test confirmed the absence of a statistically significant difference in satisfaction levels between the two cohorts (p > 0.05), indicating a consistently positive reception.

**Figure 1:**
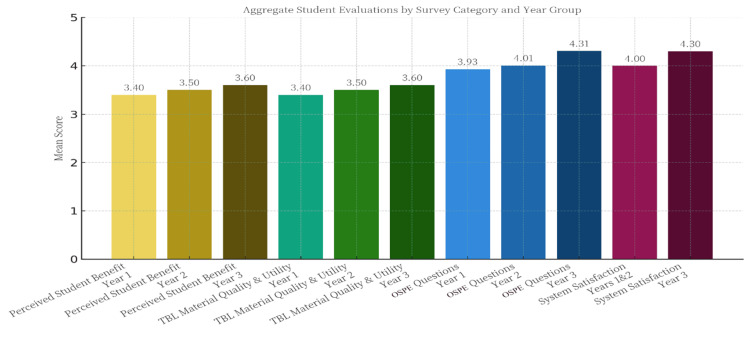
Average students’ evaluation by survey categories and year group

Strong preference among students with comparable experience (third-year): Third-year students (n = 114), who had experienced both the new system and the previous system, which lacked iRAT/tRAT assessments and OSPE integration, as it was limited only to case discussion, showed an exceptionally strong preference for the new format. The mean score for this cohort was 4.30 (SD = 1.05), with 88% of students choosing "Agree" or "Strongly Agree." Qualitative feedback from this group was particularly insightful, with responses frequently citing improvements in structure and clinical relevance. For example, one senior student stated, "The new TBL is a considerable improvement”. The old system was case discussions; while this really challenges our knowledge and preparation, making the discussion more meaningful." This finding constitutes a direct and convincing endorsement of the cohort, “third year students”, able to conduct a direct comparison (Figure 1).

Qualitative and institutional feedback

The innovative re-engineered multidisciplinary OSPE-integrated TBL model and all accompanying multidisciplinary scenarios were authored by the TBL committee chair. Prior to implementation, all authored materials underwent a rigorous validation process by a multidisciplinary faculty panel, ensuring content accuracy, relevance, and alignment with educational objectives. For this innovation, the chair received an academic citation.

Further external validation was provided by the National Center for Academic Accreditation and Evaluation (NCAAA). In their official 2016 accreditation report to the college program, the NCAAA's International Accreditation Review panel, composed of recognized experts in medical education, formally commended the TBL model. The panel's commendation was based on a comprehensive evaluation process that included direct observation of two complete sessions of the modified TBL, a thorough review of the entire application and implementation documentation, and a rigorous assessment against established accreditation standards and best practices in medical education. This formal evaluation underscores the pedagogical value and innovative nature of this integrated TBL model.

## Discussion

This study aimed to evaluate the effectiveness of a re-engineered, multidisciplinary OSPE-integrated TBL model at a Saudi medical school. Our re-engineered TBL model incorporated several key modifications to the conventional approach, as summarized in Table 1. Our results demonstrate that this innovative approach to collaborative learning resulted in substantial improvements in student participation, collaboration, and satisfaction, consistent with the findings of numerous international studies [3,38-41]. Course-specific analyses revealed consistent trends in performance improvement and demonstrated that collaborative learning significantly mitigated initial performance disparities.

Our results suggest that TBL may have an equalizing “leveling” effect on student performance, particularly during the early years of medical training. By reducing gender- and course-related performance differences, TBL effectively offset content-related challenges or difficulties through collaborative problem-solving. This "leveling effect" was particularly pronounced in conceptually challenging foundational courses, suggesting that collaboration is particularly beneficial where individual disparities are greatest. The substantially smaller range of team-based scores compared to individual assessments further reinforces this interpretation.

A significant finding of this study is the multidimensional effect of TBL-based collaboration on gender achievement gaps. Initially, female students consistently outperformed their male counterparts on individual readiness assurance tests (iRATs). However, this achievement gap narrowed considerably on team-based readiness assurance tests (tRATs), particularly during the early academic years. This phenomenon, which we term the "collaborative lift," suggests that a team-based environment fosters a more balanced academic context, an observation consistent with previous studies linking the implementation of TBL to a reduction in gender-based performance gaps [39,42].

A deeper analysis of the collaborative lift reveals a complex and dynamic impact on gender-based performance gaps across year levels. Initially, while all students benefited from teamwork, boys demonstrated proportionally greater gains from team collaboration, thereby reducing the initial achievement gap observed on iRATs. The most likely mechanism is a two-way knowledge exchange, in which higher-performing students enhance team performance by explaining concepts and simultaneously reinforcing their own understanding through instruction, a phenomenon known as the “protégé effect” [43].

However, this equalizing effect was not sustained and, crucially, appeared to reverse later. In more complex third-year courses, team-based assessments began to amplify, rather than attenuate, initial performance differences. We hypothesize that as content complexity increases, team outcomes are disproportionately influenced by the contributions of the highest-performing members of that cohort, often female students. This finding is consistent with research showing that, for complex strategic tasks, the most experienced members more heavily influence teams [44]. This finding cautions against viewing TBL as a one-size-fits-all solution and highlights that the effectiveness of collaboration is highly dependent on students' developmental level and the difficulty of the material. Therefore, educators must tailor strategies for team composition and facilitation, particularly as students progress to more advanced curricular stages.

The steady increase in individual assessment scores and the reduction in year-to-year variability suggest effective vertical integration and knowledge building across the curriculum. The more modest growth in team-based scores compared to individual scores indicates that collaborative learning provides proportionally greater benefits to students in the early years, compensating for their initial knowledge gaps. As students gain proficiency, the marginal benefit of team collaboration diminishes, a principle consistent with the expertise reversal effect, whereby instructional support useful to novices may become less effective for experts [45]. Nonetheless, team-based learning continues to provide significant benefits, even for advanced students.

In conventional team-based learning (TBL) models, peer assessment is considered a key mechanism for fostering accountability and effective teamwork [46,47]. However, much research indicates that its application can be culturally challenging and counterproductive due to a reluctance to provide critical feedback [8,19,26,32,42,48,49]. This far-reaching challenge is particularly reflected in the Saudi context. A study conducted at King Abdulaziz University by Atwa and Al Rabia (2014) found that although students perceived peer assessment positively in principle, its practical application was fraught with challenges. Nearly half of students (45%) avoided conducting peer assessments, and those who did often provided only general, uncritical feedback to avoid causing embarrassment or interpersonal conflict [50]. These local findings strongly corroborate the broader literature, which shows that in cultural contexts that prioritize social harmony, peer criticism can lead to artificially inflated scores, failing to provide true accountability [17,19,22]. It can also generate excessive anxiety if not expertly managed [51-53].

Therefore, our re-engineered TBL model intentionally omitted formal peer assessment, a strategic adaptation aimed at improving its feasibility and acceptance in our context. To compensate, we incorporated alternative accountability mechanisms, including more in-depth instructor feedback and structured team discussions. Our results confirm the effectiveness of this approach: the modified model maintained high levels of engagement and satisfaction (mean scores between 4.0 and 4.3 across all three years), while demonstrating significant learning outcomes. This adaptation addresses documented cultural and psychological barriers and contributes a context-specific framework for collaborative learning. Far from being a limitation, our approach reflects a deliberate, evidence-based adaptation to our specific educational environment, grounded in both local and international research findings.

Learning styles influenced the results of our study, which is consistent with the findings of Senol (2017), who demonstrated that different learning preferences significantly influence study time and academic performance. As Senol noted, "understanding students' learning styles would benefit to detective of productive study duration for lesson so effective working time on learning style increases academic achievement" [29]. This observation is particularly relevant in light of our recommendation to pilot a summative (graded) TBL. Implementing summative TBL offers flexibility to accommodate different learning styles by allowing students to approach the material through multiple modalities: concrete experience for accommodators, reflective observation for assimilators, abstract conceptualization for convergers, and active experimentation for divergers. By incorporating graded TBL material, we can enhance personalization for learners, as different assessment methods appeal to different learning preferences. Research shows that summative TBL improves students' motivation and learning outcomes, which can be partly explained by its ability to engage students in various aspects of their learning style, especially during online learning, where flexibility in pedagogical approaches becomes even more essential [38,54,55].

By fostering teamwork, presentation skills, and academic curiosity, these modules not only deepen subject-specific knowledge but also cultivate essential interpersonal and professional skills, as evidenced by student feedback highlighting improvements in communication, collaboration, and applied learning. Our findings are consistent with recent research by Ogut et al. (2025), who also found that cross-sectional anatomy modules significantly improved knowledge integration (74%, p = 0.004) and teamwork skills (9%, p = 0.025), with students particularly appreciating these modules for their ability to connect theoretical concepts with practical applications. Their findings reinforce the notion that specialized study modules provide a multidimensional learning experience that goes beyond content knowledge to develop essential collaboration and communication skills for future healthcare professionals [56].

The abandonment of this innovative TBL model highlights the sensitivity of pedagogical innovations to logistical challenges and changes in leadership within the correspondence learning committee. Despite its demonstrated pedagogical effectiveness, institutional factors, including scheduling conflicts and competing curricular priorities, ultimately led to the model's abandonment, particularly due to its formative and low-risk nature. This experience highlights a well-documented reality of medical education reform: evidence of pedagogical value alone is often insufficient to ensure sustainability without corresponding administrative support and infrastructure harmonization [57]. Despite these challenges, the feedback from our study, both in terms of quantitative satisfaction rates and thematic analysis of qualitative comments, consistently highlighted the value and effectiveness of the TBL approach, suggesting that the fundamental pedagogical principles remain valid even when institutional implementation proves challenging.

The study revealed an overall positive perception of the TBL model across all student cohorts, with notable consistency across genders. Of particular interest is the trend toward increasing appreciation of the TBL system with seniority. Third-year students reported higher satisfaction scores than their first-year peers, suggesting that as students gain context and clinical experience, they develop a greater appreciation for the integrative and practical nature of TBL. This finding is consistent with other longitudinal studies of collaborative learning implementation, which have also reported increased student satisfaction over time [7]. This developmental progression in student attitudes parallels the observed change in performance, reinforcing the need for year-specific adaptations in collaborative learning approaches.

Strengths

This observational study demonstrates significant collaborative engagement across all courses, with outcomes based on the documented use of authentic clinical reasoning tasks. The effectiveness of the methodology is further substantiated by external validation from the international accreditation body responsible for reviewing the whole program with curriculum effectiveness. In its official 2016 NCAAA accreditation report, the committee commended the Qassim University College of Medicine for "an effective curriculum that uses a variety of instructional formats, including PBL and TBL, which generates a high level of student engagement and enthusiasm." This official feedback validates the core TBL approach, central to our study. Furthermore, the effective integration of OSPE elements is supported by high satisfaction ratings systematically collected from participants.

Limitations

However, the results are limited by their reliance on formative rather than summative outcome measures. The single-institution context limits generalizability, and further multi-center studies with robust outcome data are needed to fully establish the approach's impact on educational equity and the utility of summative assessment with psychometric analysis and longitudinal tracking of clinical performance indicators. Furthermore, the lack of peer assessment, while a necessary cultural adaptation, may be a limitation that future research should address.

Future directions and recommendations

Based on our findings, we offer several practical recommendations for educational design: 1. Integrate team-based learning (TBL) early in the curriculum to maximize the benefits of collaboration and address performance disparities; 2. Carefully consider team composition, particularly in advanced courses where performance stratification may occur; 3. Leverage TBL specifically for conceptually complex subjects where collaborative problem-solving offers the greatest benefits; 4. Develop culturally sensitive strategies to effectively integrate peer assessment in similar environments.

Successful implementation requires careful planning, resource allocation, faculty development, and alignment with existing assessment structures to maximize the positive impact of this innovative approach [2,58].

Future implementation

The upcoming institutional colleges merger presents a significant opportunity to revive and refine this innovative TBL model. By incorporating the lessons learned from this study regarding team composition, developmental progression, and cultural adaptation, the merged colleges could implement a more sustainable and effective collaborative learning framework. This transition period offers a valuable opportunity to overcome previous logistical obstacles while preserving the pedagogical benefits demonstrated in this study. Establishing clear administrative support structures, dedicated faculty development programs, and integrated summative assessment systems will be essential to ensure the long-term sustainability of this innovative and promising pedagogical approach.

## Conclusions

Our multidisciplinary TBL model, incorporating OSPE-type assessments, shows promising results for pre-clinical medical education. Collaborative learning environments appear to improve student engagement and may reduce gender-based performance disparities during the early years of preclinical training, although this effect diminishes in advanced courses. While student satisfaction data and themes identified in stakeholder feedback confirm the pedagogical value of this approach, these results should be interpreted with caution in the absence of comparative control groups.

As the Qassim University medical schools’ curricula merger approaches (2025-2026), piloting a summative version of this model represents a strategic opportunity to build on these initial findings. Success will depend on addressing key implementation challenges: faculty development in multidisciplinary facilitation, summative assessments, and alignment with institutional frameworks. Future evaluations should include control groups and longitudinal tracking of clinical performance indicators to definitively establish the impact of the model on educational outcomes and graduate preparedness.

## Appendices

The new TBL system feedback questionnaire

Introduction and Consent

Thank you for participating in this survey. Your feedback is essential to the continuous improvement of our system. This survey is intended to understand your perception of the new Team-Based Learning (TBL) system. All responses are confidential and anonymous and will be used for quality improvement, research, and evaluation of the TBL system. Please answer each question as honestly as possible. Participation is optional, and you can skip a question or stop the survey at any time.

Please answer each question as honestly as possible.

Part A: Participant Information

**Table 7:** Part A: Participant Information

Gender:	
Male	
Female	
Year level	
First	
Second	
Third	

Part B: TBL Experience

Part C: Overall System Evaluation

Please indicate your level of agreement with the following statement.

Evaluation

Instructions: Please indicate your level of agreement with the following statements using the scale below.

1 = Strongly Disagree | 2 = Disagree | 3 = Neutral | 4 = Agree | 5 = Strongly Agree

**Table 8:** Part B: TBL Experience Evaluation

#	Domain 1: Perceived Learning and Skills Development		1	2	3	4	5
Q1	The team-based format helps me learn the course content more effectively than studying alone.		O	O	O	O	O
Q2	Team discussions have improved my ability to analyze and solve complex problems.		O	O	O	O	O
Q3	Working in a team has helped me develop effective collaborative skills.		O	O	O	O	O
Q4	Teamwork has helped me identify my knowledge deficiencies.		O	O	O	O	O
#		Domain 2: Quality of TBL Materials and Session Design	1	2	3	4	5
Q6		The TBL scenarios covered topics that required deeper understanding.	O	O	O	O	O
Q7		Discussions with my colleagues in the TBL cases scenarios helped me clarify concepts that were confusing to me.	O	O	O	O	O
Q8		The TBL scenarios provided sufficient information for analysis and diagnosis.	O	O	O	O	O
Q9		The length of the TBL scenarios was adequate.	O	O	O	O	O
Q10		The Readiness Assurance Test (RAT) questions were directly related to the TBL scenarios.	O	O	O	O	O
Q11		The integration of OSPE-type questions improved my training and understanding.	O	O	O	O	O
Q12		The time allocated for the RATs and team discussions was adequate.	O	O	O	O	O

Part C: Overall System Evaluation

Please indicate your level of agreement with the following statement.

**Table 9:** Part C: Overall System Evaluation

	For Third-Year Students	1	2	3	4	5
17	I prefer the current TBL system over the one used in previous years.	O	O	O	O	O
	For First- and Second-Year Students	1	2	3	4	5
17	Overall, I am satisfied with the current TBL system.	O	O	O	O	O

Part D: Additional Comments

Please provide us with any additional comments about your experience with the new TBL system in the space below.
